# Radiological and histopathological correlations in oligodendroglioma: a comprehensive case report

**DOI:** 10.11604/pamj.2024.49.73.45324

**Published:** 2024-11-12

**Authors:** Shivali Kalode, Sarang Banait

**Affiliations:** 1 Department of Pathology, Jawaharlal Nehru Medical College, Datta Meghe Institute of Higher Education and Research, Sawangi (Meghe), Wardha, Maharashtra, India,; 2 Indira Gandhi Government Medical College and Hospital, Nagpur, Maharashtra, India

**Keywords:** Oligodendroglioma, CT scan, histopathology, psammoma bodies, case report

## Abstract

Oligodendroglial tumors are rare tumors that constitute part of the neuroepithelial tumors of the central nervous system. A diffuse, low-grade astrocytoma (WHO grade II), oligodendrogliomas are typically encountered in adults, with children under the age of 15 accounting for about 25% of cases. Although they can arise anywhere in the central nervous system, oligodendrogliomas typically occur in the cerebral white matter. Radiotherapy is the main mode of treatment since surgical intervention is limited to the role of biopsy and management of secondary effects, due to the deep brain location of the lesion and the complexity of the involved structures. Here, we are reporting a case of an 11-year-old male, who presented with a complaint of headache and underwent contrast-enhanced computed tomography (CECT) that showed a heterogenous lesion with evidence of calcification and surrounding edema compressing the third ventricle. Oligodendroglioma was diagnosed with histopathological correlation which further confirmed the diagnosis. The patient then underwent chemotherapy and radiotherapy as adjuvant therapies. A patient's prognosis is typically bad in rare occurrences of diffuse white matter spread oligodendroglioma, which can have a significant effect on neurological health.

## Introduction

Oligodendroglial tumors are rare tumors that constitute part of the neuroepithelial tumors of the central nervous system. The World Health Organization (WHO) classifies oligodendroglial tumors into two groups: grade II oligodendroglioma and grade III (anaplastic) oligodendroglioma [1]. It is seen mainly in adults, with approximately 25% of them involving children under the age of 15 [2]. While the majority of histologic tumor subtypes are germ cell and pineal cell tumors, this region can give rise to more than 17 distinct tumor types [3]. Cancers with IDH mutations generally occur more frequently in the frontal lobe and spread across the midline than cancers without these deletions, which are more frequently observed in the temporal lobe and insula [4]. Additionally, germline CHEK2 mutations have been found in primary glioblastomas and medulloblastomas. As of right now, there is no concrete evidence linking CHEK2 to malignancies of the central nervous system [5]. Brain tumors have been linked to several genetic diseases, such as Lynch Syndrome (LS), which is inherited autosomally dominantly. Brain tumor incidence in LS families was found to be 3.35% by the age of 85, compared to 0.47% in the overall population, in a study comparing the incidence of brain tumors in LS families with that of the general population. Brain imaging may be recommended for certain LS patients as early discovery could result in routine observation and early intervention [6].

## Patient and observation

**Patient information:** an 11-year-old male came to the outpatient department of neurosurgery with the chief complaint of headache which had gradually developed over the previous two years. The patient also has a significant medical history of seizures occasionally. There was no history of trauma and other history was noncontributory.

**Clinical findings:** on physical examination, all other findings were unremarkable except for cognitive impairment.

**Timeline of the current episode:** headache and occasional seizures for 2 years.

**Figure 1:**
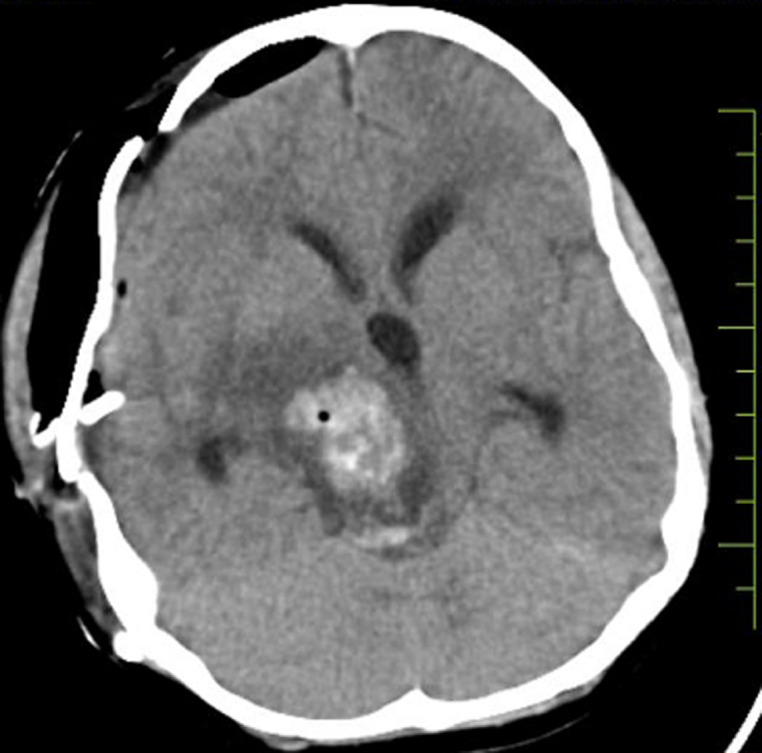
a well-defined irregular shape heterogenous lesion in the right thalamic region

**Figure 2:**
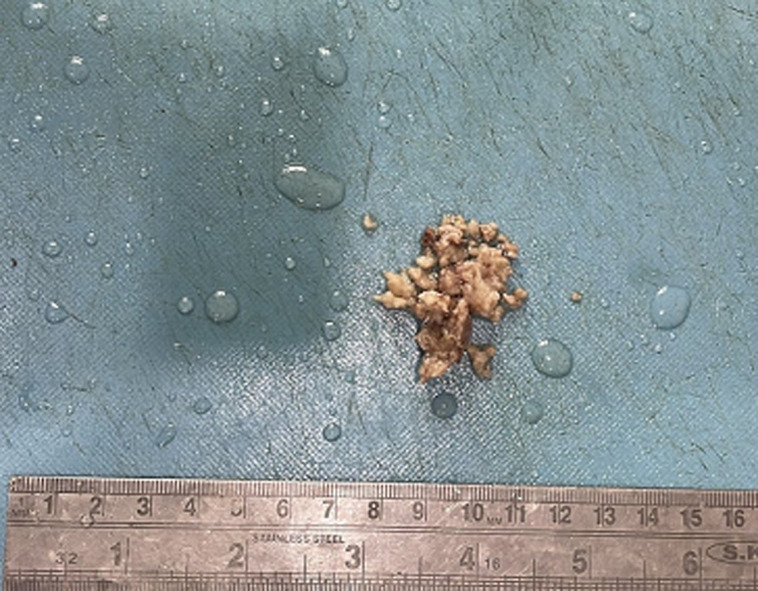
gross image-excised specimen of thalamic tumor

**Figure 3:**
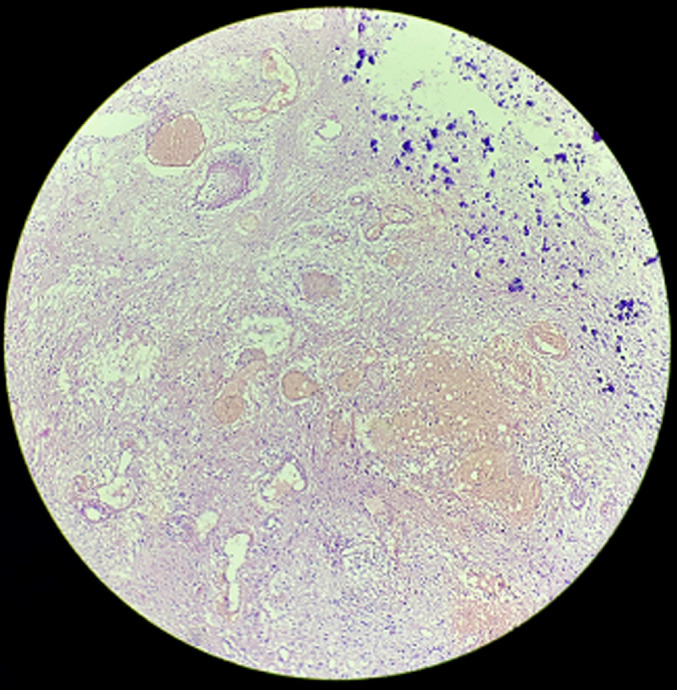
microscopic examination [H&E,20X]- uniform round nuclei and perinuclear halo in tumor cells arranged into diffuse fashion and contain many psammoma bodies

**Diagnostic assessment:** contrast-enhanced computed tomography head showed a well-defined irregular shape heterogenous lesion noted in the right thalamic region with evidence of calcification and surrounding perilesional edema. The lesion shows a mass effect in the form of compression of the third ventricle (Figure 1). Based on this report the diagnosis of neoplastic etiology likely oligodendroglioma was considered. Following this report, the excised specimen of the thalamic tumor was sent to the histopathology section in the department of pathology. Grossly, the excised specimen of the thalamic tumor measured 2 x 1 x 1 cm (Figure 2). Microscopic examination at 20X low power view showed uniform round nuclei and perinuclear halo in tumor cells arranged diffusely and containing many psammoma bodies (Figure 3).

**Diagnosis:** histopathological findings confirmed the diagnosis of oligodendroglioma.

**Therapeutic interventions:** the patient underwent adjuvant chemotherapy and radiotherapy.

**Follow-up and outcome of interventions:** the patient was advised for regular follow-up to rule out recurrence and metastasis.

**Patient perspective:** the patient was satisfied with the diagnosis and treatment.

**Informed consent:** the patient gave informed consent to publish this case report and any related photos.

## Discussion

Only 1% to 1.5% of brain tumors are primary thalamic gliomas, and bilateral thalamic tumors are considerably less common. Although they can vary from grade I to IV astrocytomas, they are often diffuse, low-grade astrocytomas (WHO grade II). The involvement of surrounding deep nuclei and white matter tracts, in particular the internal capsule's corticospinal tracts, as well as the obstructive effects of the cerebrospinal fluid circulation, all influence the clinical presentation, which varies [2]. They are predominantly tumors of adulthood, with a peak incidence between the fourth and sixth decades of life [3]. Oligodendrogliomas are typically of low density on nonenhanced CT, low signal intensity on T1-weighted, and high signal intensity on T2-weighted MRI. Imaging characteristics that are typical of oligodendrogliomas include a cortical-subcortical location, heterogeneously elevated signal intensity on T2-weighted sequences, an unclear boundary, and coarse calcification, which can be detected in up to 90% of patients [4]. There are not many alternatives for treating oligodendroglioma. Typically, the first surgery is followed by chemotherapy and radiation therapy after the procedure [5].

## Conclusion

Although oligodendroglial tumors are rare tumors that constitute part of the neuroepithelial tumors of the central nervous system, we are reporting a rare case presentation in the 11-year-old male who underwent CECT and was diagnosed with a lesion of neoplastic etiology likely oligodendroglioma and was further confirmed on histopathology. Contrast-enhanced computed tomography and MRI scans are essential along with biopsy and histopathological correlation in the definitive diagnosis and follow-up. The pros and cons of surgery, neuro-oncological treatments (chemotherapy and radiation), and imaging surveillance in these kinds of situations are still up for debate. In this case, the patient underwent adjuvant chemotherapy and radiotherapy.
